# Supplementary material for: From sequence to enzyme mechanism using multi-label machine learning
Source: BMC Bioinformatics. 2014 May 19;15:150. doi: 10.1186/1471-2105-15-150 (PMC4229970; doi:10.1186/1471-2105-15-150)
Supplement: Additional file 2 — Java code of ml2db. Additional file ml2db_code.tar.gz contains the Java source code to run the multi-label machine learning experiments and save the results to database. The code’s Javadoc is included. [file 1471-2105-15-150-S2.zip › additional file 2/ml2db/ml2db/doc/main/ml2db/weka/DataSetChecker.html]

DataSetChecker


JavaScript is disabled on your browser.


- Overview
- Package
- Class
- Use
- Tree
- Deprecated
- Index
- Help

- Prev Class
- Next Class

- Frames
- No Frames

- All Classes

- Summary:
- Nested |
- Field |
- Constr |
- Method

- Detail:
- Field |
- Constr |
- Method


main.ml2db.weka

## Class DataSetChecker

- java.lang.Object
- - main.ml2db.weka.DataSetChecker

- ---

    

  ```
  public class DataSetChecker
  extends java.lang.Object
  ```

  Checks that all data (or file paths) needed to build the ARFF file are
  available

  Version:
  :   3 Sep 2010

  Author:
  :   Luna De Ferrari ldf(at)st-andrews.ac.uk

- - ### Constructor Summary

    Constructors

    Constructor and Description

    `DataSetChecker(DataSetManager manager)`
  - ### Method Summary

    Methods

    | Modifier and Type | Method and Description |
    | --- | --- |
    | `boolean` | `dataMapsAreCorrect(InstAttValueMap instanceAttributeMap, InstAttValueMap instanceClassMap)` public boolean initialisationIsCorrect() { return m\_initialisationIsCorrect; } public void setInitialisation(boolean isCorrect) { m\_initialisationIsCorrect = isCorrect; } |
    | `ArffPropsFromFile` | `getArffProperties()` |
    | `DataSetManager` | `getManager()` |
    | `java.util.Properties` | `getProperties()` |
    | `boolean` | `propertiesAreCorrect()` |

    - ### Methods inherited from class java.lang.Object

      `equals, getClass, hashCode, notify, notifyAll, toString, wait, wait, wait`

- - ### Constructor Detail


    - #### DataSetChecker

      ```
      public DataSetChecker(DataSetManager manager)
      ```
  - ### Method Detail


    - #### dataMapsAreCorrect

      ```
      public boolean dataMapsAreCorrect(InstAttValueMap instanceAttributeMap,
                               InstAttValueMap instanceClassMap)
      ```

      public boolean initialisationIsCorrect() { return
      m\_initialisationIsCorrect; }
      public void setInitialisation(boolean isCorrect) {
      m\_initialisationIsCorrect = isCorrect;
      }


    - #### getArffProperties

      ```
      public ArffPropsFromFile getArffProperties()
      ```


    - #### getManager

      ```
      public DataSetManager getManager()
      ```


    - #### getProperties

      ```
      public java.util.Properties getProperties()
      ```


    - #### propertiesAreCorrect

      ```
      public boolean propertiesAreCorrect()
      ```


- Overview
- Package
- Class
- Use
- Tree
- Deprecated
- Index
- Help

- Prev Class
- Next Class

- Frames
- No Frames

- All Classes

- Summary:
- Nested |
- Field |
- Constr |
- Method

- Detail:
- Field |
- Constr |
- Method
